# Development and preliminary evaluation of an online educational video about whole-genome sequencing for research participants, patients, and the general public

**DOI:** 10.1038/gim.2015.118

**Published:** 2015-09-03

**Authors:** Saskia C. Sanderson, Sabrina A. Suckiel, Micol Zweig, Erwin P. Bottinger, Ethylin Wang Jabs, Lynne D. Richardson

**Affiliations:** 1 Department of Genetics and Genomic Sciences, Icahn School of Medicine at Mount Sinai, New York, New York, USA; 2 Institute of Personalized Medicine, Icahn School of Medicine at Mount Sinai, New York, New York, USA; 3 Department of Emergency Medicine, Icahn School of Medicine at Mount Sinai, New York, New York, USA

**Keywords:** informed decision making, patient education, public education, whole-genome sequencing

## Abstract

**Background:**

As whole-genome sequencing (WGS) increases in availability, WGS educational aids are needed for research participants, patients, and the general public. Our aim was therefore to develop an accessible and scalable WGS educational aid.

*Genet Med* **18** 5, 501–512.

**Methods:**

We engaged multiple stakeholders in an iterative process over a 1-year period culminating in the production of a novel 10-minute WGS educational animated video, “Whole Genome Sequencing and You” (https://goo.gl/HV8ezJ). We then presented the animated video to 281 online-survey respondents (the video-information group). There were also two comparison groups: a written-information group (*n* = 281) and a no-information group (*n* = 300).

*Genet Med* **18** 5, 501–512.

**Results:**

In the video-information group, 79% reported the video was easy to understand, satisfaction scores were high (mean 4.00 on 1–5 scale, where 5 = high satisfaction), and knowledge increased significantly. There were significant differences in knowledge compared with the no-information group but few differences compared with the written-information group. Intention to receive personal results from WGS and decisional conflict in response to a hypothetical scenario did not differ between the three groups.

*Genet Med* **18** 5, 501–512.

**Conclusions:**

The educational animated video, “Whole Genome Sequencing and You,” was well received by this sample of online-survey respondents. Further work is needed to evaluate its utility as an aid to informed decision making about WGS in other populations.

*Genet Med* **18** 5, 501–512.

## Background

Whole-genome sequencing (WGS) is increasingly offered to patients for research and clinical purposes.^1,2,3,4^ In the United States, several research projects involve individuals having their genomes sequenced and receiving personal results.^5,6,7,8,9^ In the United Kingdom, tens of thousands of National Health Service (NHS) patients are being enrolled to have their genomes sequenced and receive personal results in the Genomics England 100K Genome Project.^10^ Anyone with the financial means can also get their genome sequenced via the Illumina Understand Your Genome conferences.^11^

This points to a need for effective ways to help people make informed decisions about having their genomes sequenced. For a decision to be considered informed, it should be based on sufficient knowledge about the benefits, risks, limitations, and uncertainties regarding the technology and consistent with personal values.^12,13,14,15,16^ However, public understanding of genetics^17,18,19^ and related concepts is low. While one goal of genetic counseling is to help patients make more informed decisions,^20^ there are insufficient genetic counselors to meet the demand as WGS increases in use.^21^ Debates continue about how genetic results should be delivered to patients and research participants^22^; however, given the complexity of WGS, educational tools and communication aids that help people understand and think through key aspects of WGS and that are scalable (i.e., easily accessed by and administered to large numbers of individuals) would be valuable.

An understanding of WGS is associated with educational attainment,^23^ and educational efforts should be accessible to people of a range of educational backgrounds. Such materials also need to be appropriate for people from diverse racial and ethnic backgrounds.^17^ However, few educational resources provide clear and concise explanations of WGS and surrounding issues. Still fewer make this information accessible or relatable to diverse communities.

Videos with voiceover may be more accessible than written information for people with low literacy.^24,25^ Multimedia videos may lead to greater comprehension than other delivery formats.^26,27,28^ One advantage of animation in educational videos is that the characters can be made race- and ethnicity-neutral. This may help make such videos more accessible to a wider range of racial/ethnic groups.

We therefore developed a novel online 10-minute animated video about WGS with input from ethnically/racially diverse community consultants, community members, and patients. Here, we report the preimplementation process we carried out to develop the video. We also report the results from a preliminary postimplementation evaluation (i.e., after the video was developed and made publicly available) in which a convenience sample of online-survey respondents responded to the video.

Our primary aims were (i) to develop an educational animated video about WGS that was appropriate for and accessible to people from diverse backgrounds, (ii) to assess people's satisfaction with the video, and (iii) to determine whether knowledge about WGS was greater after people viewed the video. We also explored people's intention to receive personal results from WGS and decisional conflict after viewing the video. Decisional conflict is inversely associated with informed decision making^29^ and has been used in previous research studies to assess the impact of decision aids.^30,31^

Our secondary objectives were to provide preliminary data on how the video compared with being given written or no information about WGS. Specifically, we explored whether individuals directed to the video reported greater satisfaction with the information than individuals directed to written information. We also explored whether people directed to the video reported greater knowledge and less decisional conflict than individuals either directed to written information or given no information about WGS.

## Materials and Methods

### Development of the video

**Figure 1:**
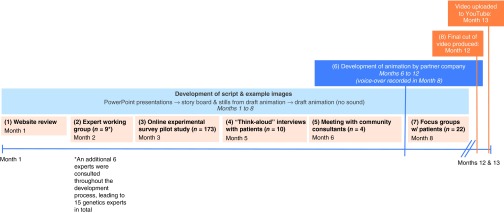
**Overview of timeline and procedures involved in the development of the video “Whole Genome Sequencing and You.”**

As **Figure 1** illustrates, the animation was developed in eight phases. The final animation was then shared directly with the public via YouTube, the video-sharing website that hosts user-generated videos (http://www.youtube.com), as well as via the website of the Icahn School of Medicine at Mount Sinai Institute of Personalized Medicine (http://icahn.mssm.edu/research/institutes/institute-for-personalized-medicine). See **Supplementary Information** online for full details. The medical school's institutional review board approved this research.

### Preliminary evaluation of the final video

To provide preliminary data evaluating the animated video postimplementation, an online survey was conducted. This allowed us to quickly and easily receive feedback on the video from several hundred people. We used an experimental study design in which the online-survey respondents who were presented with the video were randomly selected from a larger pool of 862 respondents in total. One-third (*n* = 281) of the survey respondents were randomly assigned to the video-information group, one-third (*n* = 281) to a written-information group, and one-third (*n* = 300) to a no-information group (see study flowchart in **Supplementary Figure S1** online). The purpose of the written-information group was to preliminarily explore how the video compared with information presented in text form. Participants in this group were provided with an online WGS information “pamphlet” comprising 1,138 words of written text and several basic example images (slightly less than the length of the video voiceover script, which was 1,384 words). The pamphlet had previously been developed for use in the phase 3 pilot study and therefore provided a convenient comparison, although because it was an early draft of the content it did not contain the “Making Your Decision” section that was included in the video (**Supplementary Table S1** online). 

The purpose of the no-information group was to preliminarily explore whether the video led to less decisional conflict and greater knowledge than if no information was provided. Because WGS is unfamiliar to many people, the no-information group was provided with a very brief description (four sentences comprising 81 words in total) of what WGS is so that they had sufficient understanding to answer the survey questions. The full animation script, pamphlet text, and brief description of WGS are available from the corresponding author.

The participants were recruited through Survey Sampling International (SSI; http://www.surveysampling.com), an online market research company. Typically, to recruit survey participants to their panels, SSI uses pop-up windows and banner ads on various websites, including their affiliate partners' websites, social media, and online communities. Interested individuals are invited to be on the SSI panel of participants and asked to answer a set of multiple-choice questions so that they can be characterized in terms of demographic and other descriptive variables. They are then randomly invited to complete the online surveys for which they are eligible. SSI uses a confidential identification number (which they do not share with investigators) to provide respondents with an incentive (a quarterly drawing for $25,000) to complete a survey. Every respondent who completes at least one survey during a given quarter is entered into the quarterly contest. An additional incentive is provided to participants aged between 18 and 23 years because of their relatively low response rate; individuals in this age group are offered 300 points (equivalent to $3) to complete a survey.

For the present study, participants were provided with a link to the online survey on SurveyMonkey.com. After reading the information on page 1 of the survey, participants consented by clicking on “continue.” After answering the first questions, participants were then randomized to the video-information group, the written-information group, or the no-information group. The video-information group was provided with a link to the video on YouTube and asked to click on the link, watch the video, and return to the survey. The written-information group was asked to read the online pamphlet. The no-information group was provided only with the very brief description of WGS. All were then asked to answer further questions. Full survey instruments are available from the corresponding author. The study was determined to be exempt from institutional review board approval by the Icahn School of Medicine at Mount Sinai's institutional review board.

### Measures used in the preliminary evaluation of the final video

Measures included sociodemographics, satisfaction, informed decision making (intention, decisional conflict), and knowledge (see **Supplementary Table S2** online for when each measure was administered). All measures were administered in the same format to all participants in all three groups with one exception: “satisfaction” items differed between the video-information and written-information groups in that the word “video” was replaced with “educational material” for the latter group, and these items were not administered to the no-information group because they did not receive any educational materials to evaluate.

*Sociodemographic baseline characteristics.* Gender, age, race/ethnicity, employment, annual household income, number of biological children, and education were assessed. Self-rated understanding of genetics at baseline was assessed with two items, one of which (“compared with others”) was adapted from previous research.^32^

Satisfaction. Participants' satisfaction with the video/written information was evaluated using nine items adapted from previous research.^33^ A combined satisfaction scale score was generated by calculating the mean of seven items (1 = low satisfaction, 5 = high satisfaction); in factor analysis, all seven items loaded onto a single factor with eigenvalues >0.4. Cronbach's α was 0.89, indicating good reliability. Perceived utility of the information for decision making was assessed using one item adapted from previous research.^34^

*Knowledge*. Objectively assessed knowledge: Objective knowledge was assessed using a previously published measure comprising 11 items, each with five response options, developed in the NIH ClinSeq Study.^23^ This measure was chosen because it is currently the only published measure of knowledge about WGS. In the original publication,^23^ two factors emerged in factor analysis: “sequencing limitations knowledge” (five items) and “sequencing benefits knowledge” (five items) (Cronbach's α preconsent = 0.80 and 0.70, respectively). The “lifestyle genomics” item (“*A person's health habits, such as diet and exercise, can affect whether or not their genes cause diseases*”) did not load onto either factor with eigenvalue >0.40. For each of the true statements, “strongly agree” = 2, “somewhat agree” = 1, and the other three responses = 0. False statements were reverse-scored. Each scale therefore had a possible range of 0–10 (where 10 = high knowledge). To be able to directly compare scores in our study with the original scores, we calculated both scales in accordance with the approach used in the original publication.^23^ In addition, we reported the lifestyle genomics item separately (1 = low knowledge, 5 = high knowledge).

*Self-rated knowledge:* Subjective understanding of seven WGS-related terms was measured at baseline and posteducation using a measure adapted from previously published research.^35^

*Intention and decisional conflict*. After being presented with information about a hypothetical WGS research study, participants were asked to imagine that they had been invited to take part in this study and were told they could choose to learn personal genetic results. They were then asked whether they would want to receive personal results using one item adapted from previous research.^36^ The Decisional Conflict Scale (DCS) was used to measure decisional conflict regarding this intention.^14^ The DCS is a 16-item scale scored on a 5-point scale (0 = strongly agree, 4 = strongly disagree) and is reported as one overall score and five subscales (informed, effective decision making, support, values clarity, and uncertainty). Possible DCS scores range from 0 (no decisional conflict) to 100 (high decisional conflict); scores <25 are associated with implementing decisions; scores >37.5 are associated with decision delay or feeling unsure about implementation.

*Statistical analyses*. In brief, variables were described using frequencies, means, and standard deviations. Changes over time were assessed using Wilcoxon signed-ranks tests and McNemar tests. Between-groups comparisons were calculated using χ^2^ tests, Kolmogorov-Smirnoff tests, Kruskall-Wallis tests, analyses of covariance, and binary logistic regressions. *P*-values <0.05 were considered significant. All analyses were performed using IBM SPSS 20 (Chicago, IL).

## Results

### Development of the video

*Phase 1: Review of existing online resources.* A review of existing online educational websites about genomics and WGS was conducted during the autumn of 2011. Four websites were identified that met some, but not all, of our criteria in terms of addressing genomics concepts and WGS and being easily accessible to a wide and varied audience with a range of literacy levels (see **Supplementary Information** online). After this review, an initial text outline and example images were developed.

*Phase 2: Expert working group.* After convening our expert working group and obtaining input from two additional genetics experts and four opportunistically recruited lay individuals, a first draft of the educational material text was produced. This comprised the following: (i) Genetics 101, (ii) the brief description of WGS, and (iii) explanations of seven categories of information potentially generated by WGS (genetic disorders, genetic carrier status, common disease susceptibility, pharmacogenetics, physical traits, ancestry, and DNA variants of unknown significance).

*Phase 3: Online experimental survey: pilot study.* Of the 173 individuals who completed the online experimental survey pilot study, 21% were Hispanic/Latino, 30% were African-American, 41% were non-Hispanic white, and 8% were other racial/ethnic backgrounds. Respondents were randomly assigned to a written WGS education *without* the Genetics 101 group (*n* = 56), a written WGS education *with* the Genetics 101 group (*n* = 54), or a no-information group (*n* = 63). The group that received Genetics 101 was more satisfied with the information than the group that did not receive Genetics 101 (*P* = 0.006). We therefore retained the “Genetics 101” information section. Based partially on other feedback from the survey respondents, and on the same experts who had reviewed the first draft, the content was significantly revised to include new or edited sections on genetic variation, categories of information, ancestry, DNA variants of unknown significance, limitations, and benefits.

*Phase 4: Think-aloud interviews.* Of the 10 patients who completed the think-aloud interviews, six were African-American, two were Hispanic, and two were non-Hispanic white. In the quantitative questionnaires, all participants agreed the material was easy to read. Nine felt the material was relevant to them. All were satisfied with the organization of the material. Six thought the amount of information was the right amount, whereas three felt there was too much information. All said that the pros and cons of WGS were easy to understand. Revisions were based on both quantitative and qualitative results (see **Supplementary Information** online).

*Phase 5: Meeting with community consultants.* The four community consultants from the East Harlem and Upper East Side areas of Manhattan provided important insights that led to several key changes to the educational material. For example, they indicated that the section on genetic variation between individuals needed to be explained more clearly; therefore, revisions were made to address this. See **Supplementary Information** online.

*Phase 6: Partnership with the animation company.* Once given the final script and example images, an animation company developed the animation, working in an iterative process with study investigators and responding to feedback from focus-group participants (see below).

*Phase 7: Focus groups with community members.* The three focus groups comprised 22 participants: seven African Americans, six Asians, four Hispanics, four non-Hispanic whites, and one native Hawaiian. Participants responded negatively to two sections of the video in particular. The animation company was given this and other feedback from the groups, and revisions were made. See **Supplementary Information** online.

*Phase 8: Final edit.* The final edit of the video was approved by study investigators and posted to YouTube on 8 November 2012 (see https://goo.gl/HV8ezJ). The video used minimal text; see **Supplementary Figure S2** online for still images from the final animation. **Supplementary Table S1** online summarizes the topics covered.

### Preliminary evaluation of the final animated video

*Video-information group*

**Figure 2:**
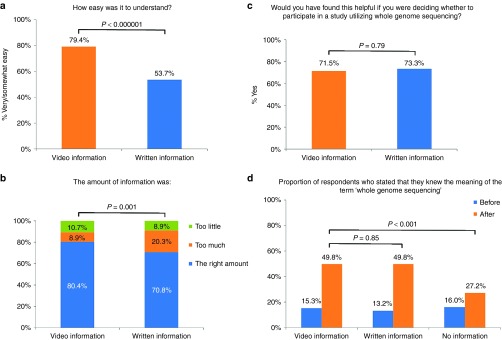
**Satisfaction and knowledge by experimental group in the postimplementation online survey**. (**a**) Proportions of participants who found the educational information easy to understand compared between the animation group and text group (χ^2^ = 41.4; df = 2; *P* < 0.000001). (**b**) Proportion of participants who found the amount of information was the right amount compared between the animation group and text group (χ^2^ = 14.66; df = 2; *P* = 0.001). (**c**) Proportion of participants who would have found the educational information helpful if deciding whether to participate in a study utilizing WGS compared between the animation group and text group (difference not significant). (**d**) Proportion of respondents who stated they knew the meaning of the term “whole-genome sequencing” before and after the educational information, compared between the animation group, text group, and minimal-information group (before: χ^2^ = 6.58; df = 4; *P* = 0.16; after: χ^2^ = 48.59; df = 4; *P* < 0.000001).

*Sociodemographic characteristics. Of the 281 respondents in the video-information group, 48.2% were female, the mean age was 44.9 years (range 18–80 yr), 29.5% were African-American, 13.2% were Hispanic, 40.6% were white, 56.2% had an annual income <$40,000, 50.0% were employed, and 36.2% had a degree (**Table 1**).* *Satisfaction.* Seventy-nine percent of respondents said the video was easy to understand (**Figure 2a**). The mean (SD) satisfaction scale score was 4.00 (0.82), indicating high overall satisfaction with the video (see **Table 2** for individual satisfaction items). The majority (80%) said the amount of information presented in the video was “the right amount” (**Figure 2b**). Seventy-two percent said they would find the video helpful if they were deciding whether to participate in a study utilizing WGS (**Figure 2c**).

*Knowledge*. Objective knowledge about the benefits of WGS and about lifestyle genomics increased significantly from preintervention to postintervention. There was no change in objective knowledge about the limitations of WGS. Self-rated understanding of genomics terms increased significantly for the terms “whole-genome sequencing” (**Figure 2d**), “genome,” “gene,” “pharmacogenetics,” and “DNA variant of unknown significance,” but not for “DNA” or “chromosome” (**Table 3**).

*Intention and decisional conflict.* The majority (71%) of respondents stated they would want to find out their personal genetic results from WGS. Mean DCS scores ranged from 29.09 to 32.47 (where 0 = no decisional conflict, 100 = high decisional conflict), suggesting moderately low levels of decisional conflict (**Table 3**).

*Comparisons between experimental groups* Sociodemographic characteristics. There were more women in the no-information group than in the video-information and written-information groups (61, 48, and 49%, respectively: *P* = 0.004; **Table 1**). Women also reported lower levels of self-rated understanding of genetics and genomic terms than men (data not shown). Gender was therefore included as a covariate in analyses comparing knowledge between the three experimental groups. Although there was a statistically significant difference in income, the median income was $20,000–$39,000 in all three experimental groups; therefore, income was not included as a covariate (**Table 1**).

*Satisfaction.* Respondents in the video-information group were more likely than those in the written-information group to report that the material was very or somewhat easy to understand (79 vs. 54% respectively; *P* < 0.000001; **Figure 2a**) and less likely to say the amount of information was “too much” (9 vs. 20%: *P* = 0.001; **Figure 2b**). However, the two groups did not differ on the satisfaction scale or perceived utility of the information for decision making (**Table 2** and **Figure 2c**).

Knowledge. For both objectively assessed knowledge about WGS and self-rated understanding of genomics terms, the video-information group and the no-information group differed significantly; knowledge increased in the video-information but not in the no-information group. There were no differences between the video-information and written-information groups (**Figure 2d** and **Table 3**).

Intention and decisional conflict. There were no between-group differences in intention to receive personal results from WGS or decisional conflict (**Table 3**).

### Factor structure and reliability of the objective knowledge measure

In addition to calculating our objective knowledge scales in accordance with the work by Kaphingst et al.^23^ to enable comparisons between studies, we examined the factor structure in our own study.^23^ As **Supplementary Table S3** online shows, we found seven items loaded on factor 1 and four on factor 2. This reflected two differences between studies. First, Kaphingst et al.^23^ found that “*Even if a person has a variant in a gene that affects their risk of a disease, they may not develop that disease*” loaded onto the “limitations knowledge” factor; in our study, this loaded with the “benefits knowledge” factor. Second, Kaphingst et al.^23^ found the lifestyle genomics item did not load on either factor. However, in our study, it loaded with “benefits knowledge.” When using Kaphingst's five items to create the “limitations knowledge” scale, we obtained Cronbach's α = 0.62 (i.e., below the 0.7 cut-off), indicating this scale did not have good reliability in our sample. However, α = 0.70 for our sample if “*Even if a person has a variant in a gene that affects their risk of a disease, they may not develop that disease*” was excluded. The “benefits knowledge” scale had good reliability in our sample regardless of whether we used Kaphingst's five items (α = 0.82) or our seven items (α = 0.80).

## Discussion

We developed and evaluated a novel online educational animated video about WGS using a mixed-methods approach including think-aloud interviews, focus groups, and online experimental surveys. Our use of these multiple methods to engage patients as critical stakeholders during preimplementation (development) and postimplementation (evaluation) of this novel WGS health-information technology was consistent with the approaches described by Hartzler et al.,^37^ who also noted that stakeholder engagement using these types of methods “is critical for successful implementations of systems and processes that can support the use of genomic information.” The preimplementation, development phase of our work with diverse patients and community members led to multiple revisions of the animation content that served to improve the quality and presentation of the information technology and make it more appropriate and accessible for the target audiences.

Among online-survey respondents who were asked to view the video in the postimplementation phase, satisfaction was high and knowledge increased. This is consistent with prior clinical genetics research, particularly cancer genetics (e.g., *BRCA1/2* testing), that has demonstrated that educational tools designed to aid understanding (e.g., written materials, interactive computer programs) can increase knowledge and elicit positive satisfaction scores.^38,39,40^ Our work builds on these early educational efforts in clinical genetic testing by expanding the focus to WGS and delivery mode to animated video.

Although decisional conflict did not differ between the groups, there were greater improvements in objective and self-rated knowledge among online-survey respondents who were directed to the video than those who received no information about WGS and some evidence of higher levels of satisfaction with the materials in the video-information group compared with the written-information group. These findings tentatively support that the video was beneficial and had some positive educational effects. Furthermore, although literacy was not assessed, the respondents were all part of an online market research panel who, by definition, had sufficiently high literacy levels to complete written questionnaires. It is possible the video will be more effective for individuals with lower literacy levels in future investigations.

Our findings have methodological implications for the measurement of WGS knowledge. The ClinSeq measure^23^ we used to assess objective knowledge about WGS is important because it is currently the only measure of its kind. To our knowledge, our study with 862 participants is the largest to date to have administered this measure. Our factor analysis and scale-reliability calculations raise questions about the measure's validity and reliability in different populations. Although this measure has provided a valuable starting point, there is a need for further work to develop a more definitive objective measure of WGS knowledge that addresses additional concepts (e.g., risks) and that is reliable and valid in multiple different populations. A greater understanding of the relationships between subjective and objective knowledge measures would also be useful.

Limitations of the present study included that the video was evaluated by online-survey respondents who may be more comfortable with online information than other audiences, and that it was not evaluated by individuals actually considering real WGS. This is important because people's responses to hypothetical scenarios are imperfect predictors of their reactions to real-world contexts.^41,42^ However, we will be able to address this in future work because we have been using the video as a communication tool during the genetic counseling in the HealthSeq project at the Icahn School of Medicine at Mount Sinai, in which 35 healthy individuals had personal genome sequencing.^43^ Additionally, we were unable to track how much of the video participants watched, or how much of the written information participants read, so we do not know whether the information was delivered as intended. Importantly, the written-information group received content that was not identical to the video content received by the video-information group. The rationale was that a “pamphlet” already existed and was convenient to use, but this presents a potential confounder between these two groups, and the comparisons between these two groups should be interpreted with caution. To accurately test the added value of the animation format over written text, further studies will be needed comparing identical information content in animated versus written delivery formats. However, the limitations are outweighed by the strengths, which include the fact that this is the first relatively short and easily accessible animated video to be developed to educate people about personal WGS and the issues surrounding this technology, with input from diverse patient and community stakeholder groups.

Finally, a critical feature of our educational aid is that it is freely available to anyone on the video-sharing website YouTube. Our intention was always that our educational aid should be useful for physicians explaining WGS to their patients in the clinical context, for researchers explaining WGS to participants in the research context, and as an educational resource for the general public. By making our video freely available in this way, we hope we are maximizing its value and usefulness in these varied contexts.

In conclusion, we developed an educational animated video about WGS that was well received by a sample of online-survey respondents. Further work is needed to evaluate its utility as an aid to informed decision making about WGS in other populations.

## Disclosure

The authors declare no conflict of interest.

## Figures and Tables

**Table 1:**
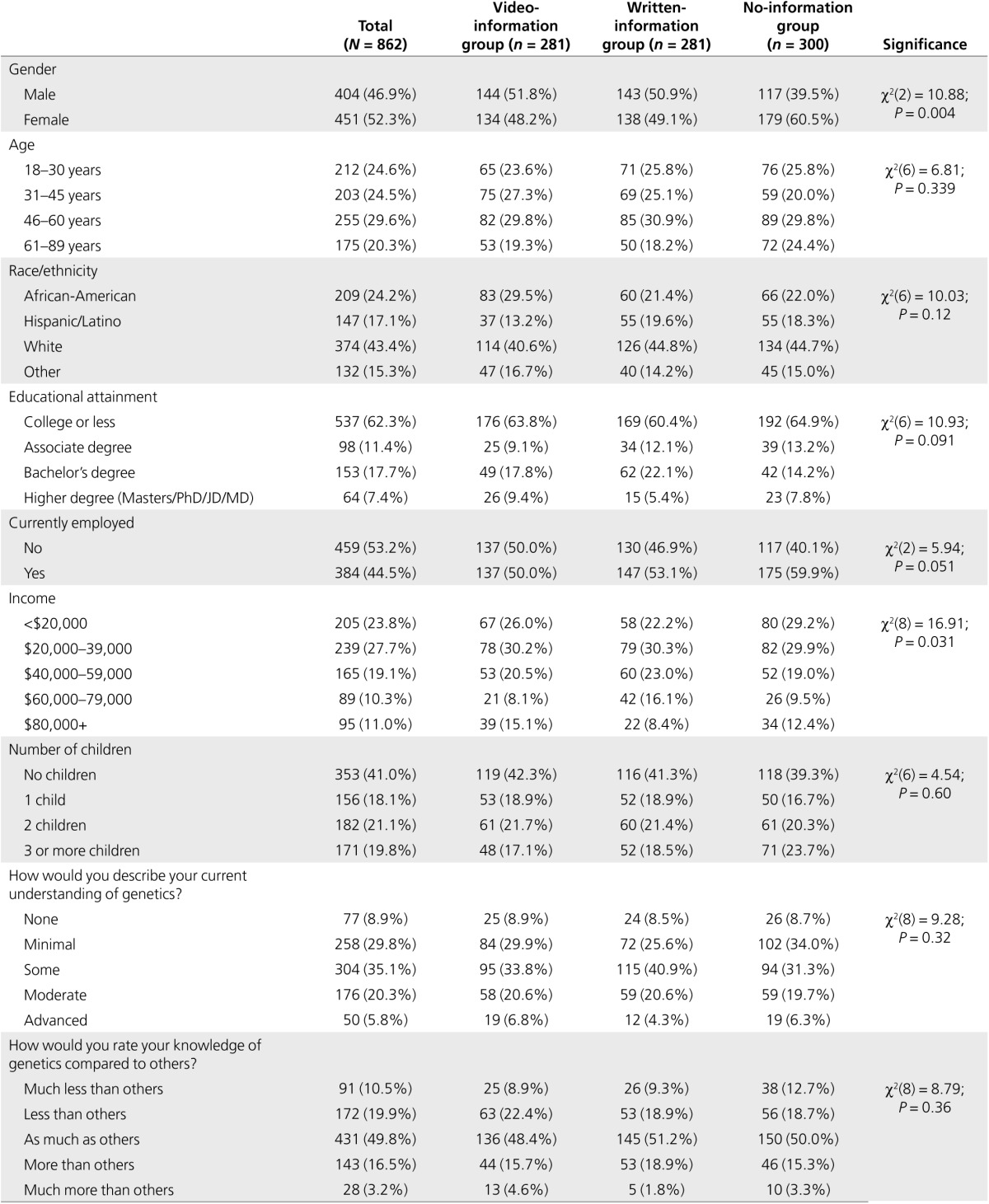
Sociodemographic characteristics and self-rated understanding of genetics at baseline by experimental group in the postimplementation online survey

**Table 2:**
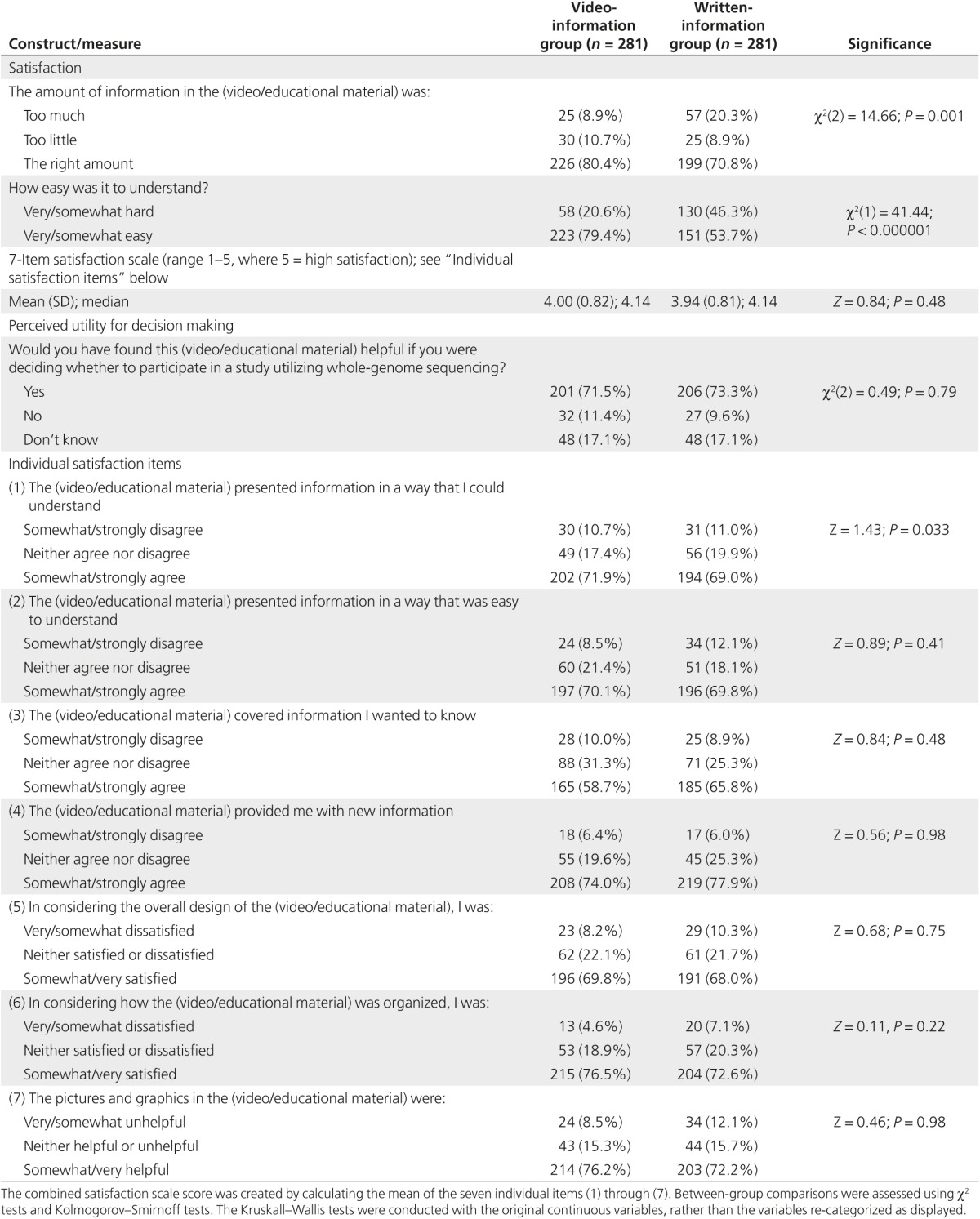
Satisfaction and perceived utility of the information for decision making compared between the video-information and written-information groups

**Table 3:**
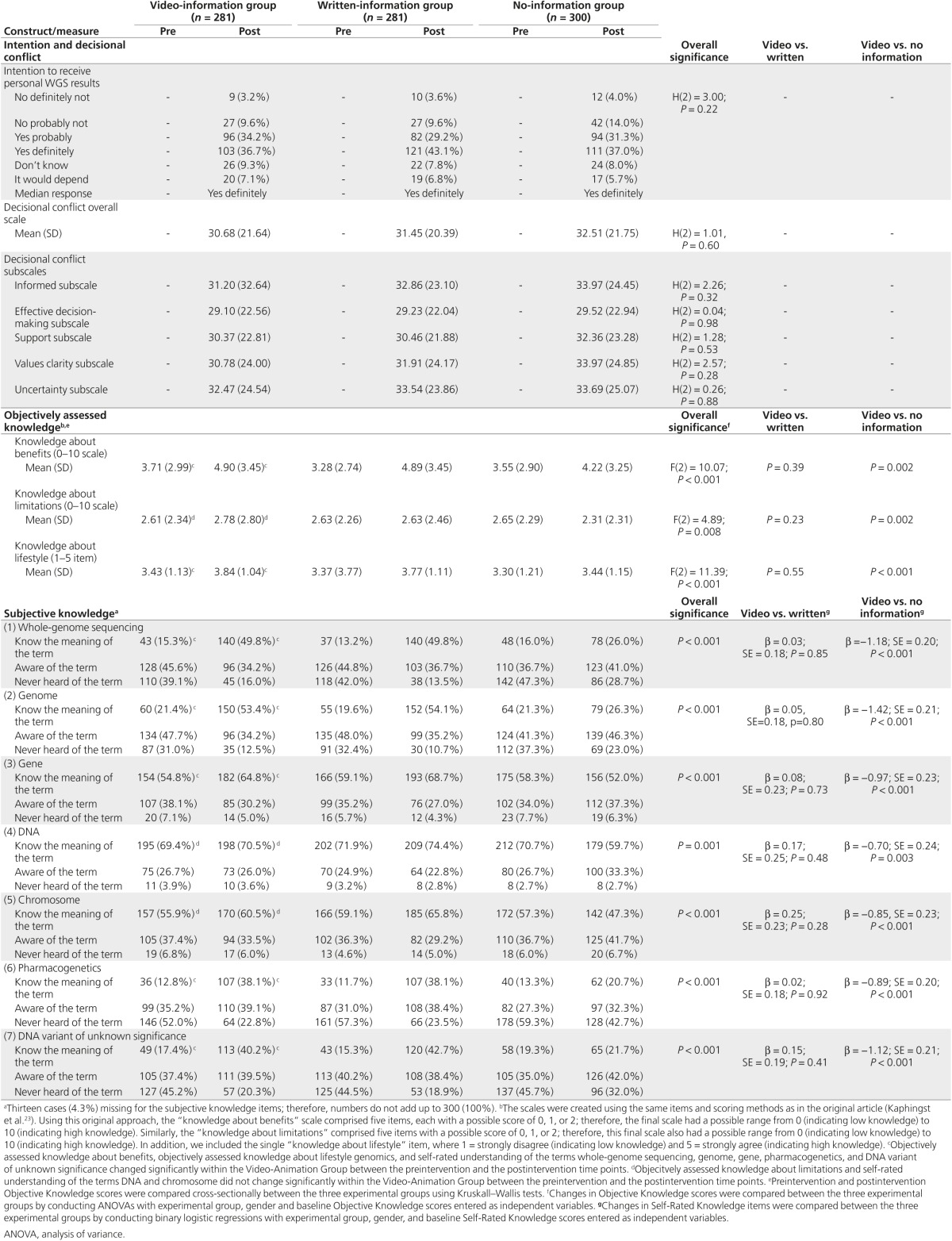
Intentions, decisional conflict, and knowledge among online-survey respondents compared between experimental groups
