# Supplementary material for: Development and preliminary evaluation of an online educational video about whole-genome sequencing for research participants, patients, and the general public
Source: Genet Med. 2015 Sep 3;18(5):501–12. doi: 10.1038/gim.2015.118 (PMC4857185; doi:10.1038/gim.2015.118)
Supplement: Supplementary Figures [file gim2015118x1.ppt]

## Slide 1
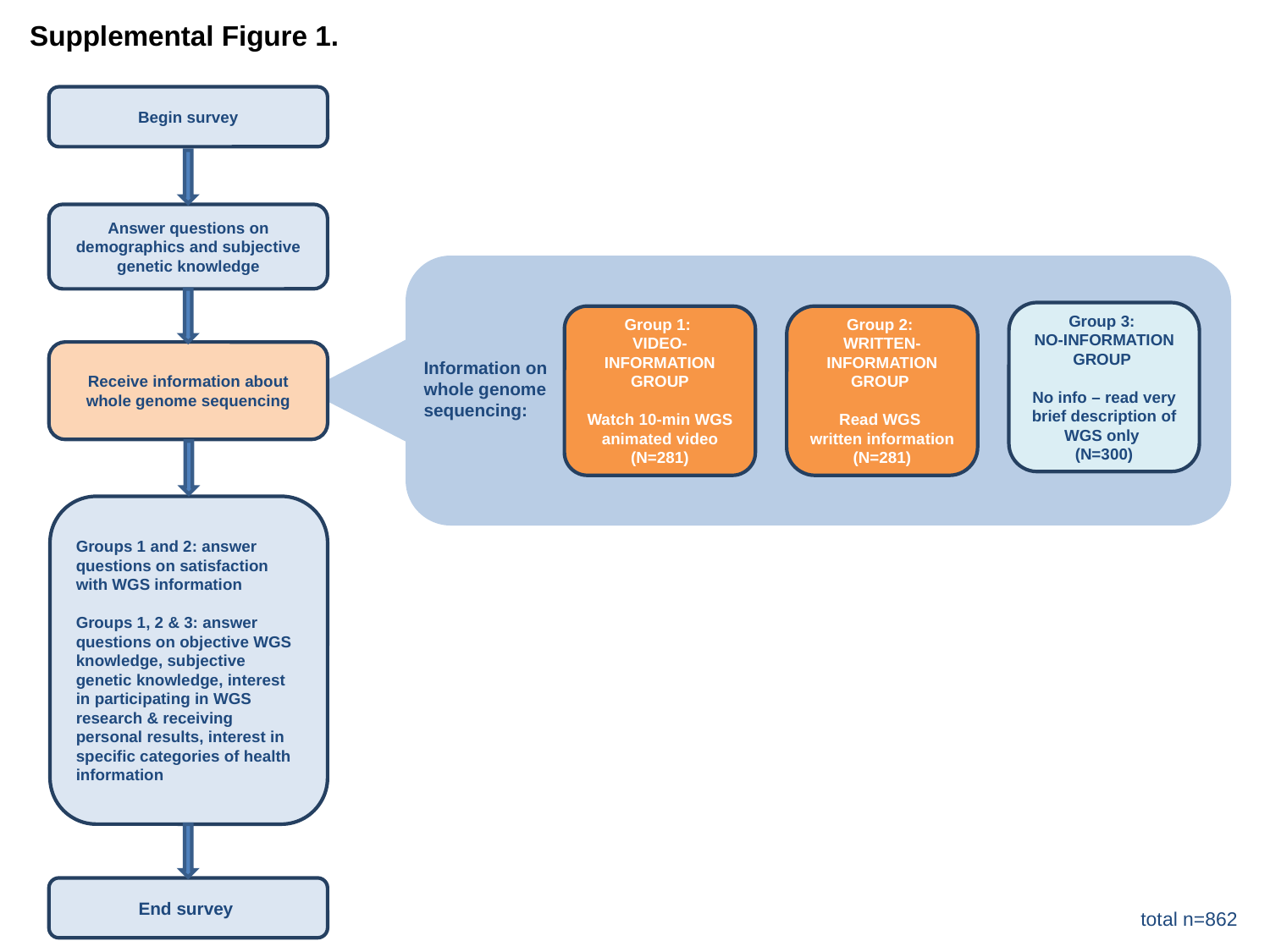

Supplemental Figure 1.
Begin survey
Answer questions on demographics and subjective genetic knowledge
Group 3:
NO-INFORMATION GROUP
No info – read very brief description of WGS only
(N=300)
Group 1:
VIDEO-INFORMATION GROUP
Watch 10-min WGS animated video (N=281)
Group 2:
WRITTEN-INFORMATION GROUP
Read WGS
written information (N=281)
Receive information about whole genome sequencing
Information on whole genome sequencing:
Groups 1 and 2: answer questions on satisfaction with WGS information
Groups 1, 2 & 3: answer questions on objective WGS knowledge, subjective genetic knowledge, interest in participating in WGS research & receiving personal results, interest in specific categories of health information
End survey
total n=862

## Slide 2
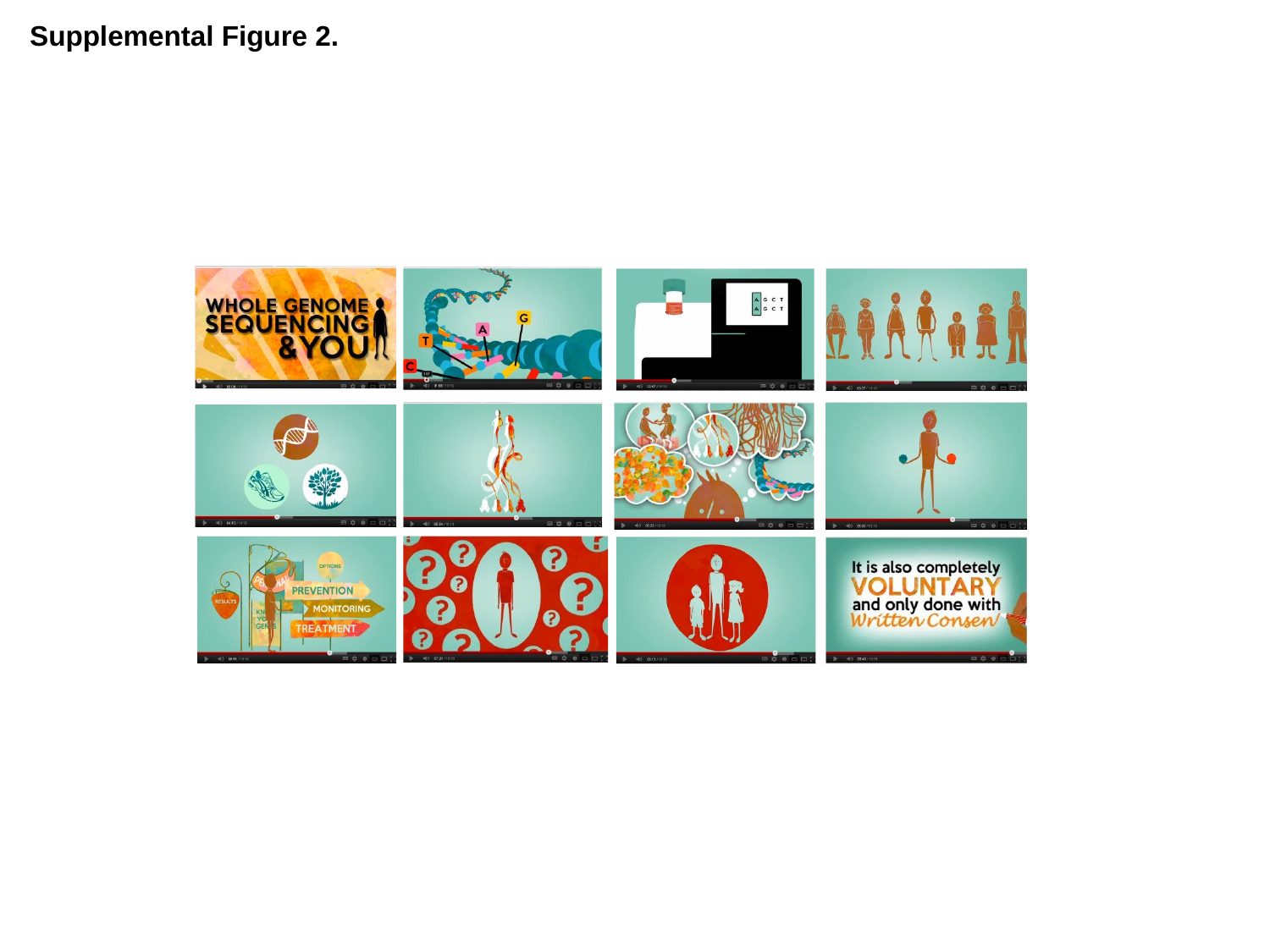

Supplemental Figure 2.
