# Supplementary material for: Development and preliminary evaluation of an online educational video about whole-genome sequencing for research participants, patients, and the general public
Source: Genet Med. 2015 Sep 3;18(5):501–12. doi: 10.1038/gim.2015.118 (PMC4857185; doi:10.1038/gim.2015.118)
Supplement: Supplementary Tables [file gim2015118x2.doc]

**Supplemental Table 1.** Sections and topics covered in the final “Whole Genome Sequencing and You” animation and the written information

| **Section** | **Animated video**  **topics** | **Written information**  **topics** |
| --- | --- | --- |
|  |  |  |
| **1. Genetics: the basics** | - Basic genetic concepts | - Basic genetic concepts |
|  | - Human genetic variation | - Human genetic variation |
| **2. What is whole genome sequencing?** | - Procedures | - Procedures |
|  | - Types of personal information that could be produced | - Types of personal information   that could be produced |
|  | - - Physical traits | - - Physical traits |
|  | - - Ancestry | - - Ancestry |
|  | - - Risk of common diseases, e.g. type 2 diabetes | - - Risk of common diseases,   e.g. type 2 diabetes |
|  | - - Risk of psychiatric disorders |  |
|  | - - Genetic disorders | - - Genetic disorders |
|  | - - Carrier status | - - Carrier status |
|  | - - Variants of unknown significance | - - Variants of unknown significance |
|  | - - Pharmacogenetics | - - Pharmacogenetics |
| **3. Making your decision** | - Benefits |  |
|  | - - Personal: prevention, monitoring, treatment |  |
|  | - - Contribute to genomics research |  |
|  | - Risks |  |
|  | - - Possible social or emotional consequences |  |
|  | - - Potential loss of privacy |  |
|  | - - Implications for family members |  |
|  | - Limitations |  |
|  | - - Interpretation of DNA sequence may not provide any information about your health |  |
|  | - - Technology is still in its infancy |  |
|  | - - Complexity of calculating an individual’s disease risk |  |
|  | - Privacy |  |
|  | - - Genome sequencing is: |  |
|  | - - Voluntary |  |
|  | - - Only done with written consent |  |
|  | - - Confidential |  |

**Supplemental Table 2. Variables assessed in the online experimental survey and the timepoints at which they were measured**

|  | **Before WGS information** | **After WGS information** |
| --- | --- | --- |
| **Background characteristics** |  |  |
| Socio-demographics | X |  |
| Self-rated understanding of genetics | X |  |
| **Primary outcomes** |  |  |
| Satisfaction with the video [written information] |  | X |
| Perceived utility of the video [written information] for decision-making |  | X |
| Objective understanding of WGS (limitations, benefits) | X | X |
| Subjective understanding of WGS (terms) | X | X |
| **Secondary outcomes** |  |  |
| Intention to receive personal results from WGS |  | X |
| Decisional conflict (DCS – multiple subscales) |  | X |
| General attitudes towards WGS research |  | X |

**Supplemental Table 3.** Factor analysis of the objective knowledge items assessed among the 862 online experimental survey participants at baseline (pre-intervention)

| **(N=862)**  **Questionnaire item** | **Factor 1**  ***(Kaphingst’s “benefits knowledge”)***^3^ | **Factor 2**  ***(Kaphingst’s “limitations knowledge”)***^4^ |
| --- | --- | --- |
| Once a variant in a gene that affects a person’s risk of a disease is found, that disease can always be prevented or cured. [false] | 0.02 | 0.76 |
| A health care provider can tell a person their exact chance of developing a disease based on the results from genome sequencing. [false] | -0.18 | 0.70 |
| Scientists know how all variants of genes will affect a person’s chances of developing diseases. [false] | -0.06 | 0.75 |
| Genome sequencing is a routine test that most people can have through their physician’s office. [false] | -0.10 | 0.61 |
| **Even if a person has a variant in a gene that affects their risk of a disease, they may not develop that disease.^1^ [true]** | **0.71** | 0.13 |
| Genome sequencing may find variants in a person’s genes that they can pass on to their children. [true] | 0.81 | -0.07 |
| Genome sequencing may give a person information about their chances of developing several different diseases. [true] | 0.84 | -0.05 |
| Genome sequencing may find variants in a person’s genes that will increase their chance of developing a disease in their lifetime. [true] | 0.84 | -0.07 |
| Genome sequencing may find variants in a person’s genes that will decrease their chance of developing a disease in their lifetime. [true] | 0.57 | -0.30 |
| Genome sequencing may find variants in a person’s genes that may determine how they respond to certain medicines. [true] | 0.52 | -0.36 |
| **A person’s health habits, such as diet and exercise, can affect whether or not their genes cause diseases.^2^ [true]** | **0.44** | -0.26 |

^1^ In ClinSeq (n=311), Kaphingst et al (2012) found that this item *(“Even if a person has a variant in a gene that affects their risk of a disease, they may not develop that disease”)* loaded on to what they labelled as the “limitations knowledge” factor: in our study, however, this is loading with the “benefits knowledge” factor.

^2^ In ClinSeq (n=311), Kaphingst et al (2012) found that this item *(A person’s health habits, such as diet and exercise, can affect whether or not their genes cause diseases”)* did not load on to either of the two factors, and so this item was excluded from their scales. However, in our study, it clearly loads with the “benefits knowledge” factor.

^3^ Scale reliability for *Limitations Knowledge Scale*: Using Kaphingst’s original 5 items to create the “limitations knowledge” scale, Cronbach’s **α=0.62** which is below the cut-off of 0.7, suggesting this scale does not have good reliability in our sample. However, Cronbach’s **α=0.70** if the item “*Even if a person has a variant in a gene that affects their risk of a disease, they may not develop that disease”* is excluded, i.e. if only the four items that load onto this factor in our factor analysis are included.

^4^ Scale reliability for *Benefits Knowledge Scale:* Using Kaphingst’s original 5 items to create the “benefits knowledge scale, Cronbach’s **α=0.82**, indicating good reliability. Using our 7 items to create the “benefits knowledge scale, Cronbach’s **α=0.80**, also indicating good reliability.

Note: The 11 items were analysed in their raw form, i.e. the original responses on the 5-point scales were used, rather than the responses having been dichotomized into “correct” or “not correct”.
