# Supplementary material for: Development and preliminary evaluation of an online educational video about whole-genome sequencing for research participants, patients, and the general public
Source: Genet Med. 2015 Sep 3;18(5):501–12. doi: 10.1038/gim.2015.118 (PMC4857185; doi:10.1038/gim.2015.118)
Supplement: Supplementary Information [file gim2015118x3.doc]

**SUPPLEMENTAL INFORMATION**

**DEVELOPMENT OF THE ANIMATION**

**Overview**

A review of existing online resources was conducted (Phase 1), and an initial content outline was drafted. An expert working group was convened (Phase 2), and the draft ideas were expanded into a first draft of the text and example images. A pilot online experimental survey study was then conducted in which 173 individuals recruited through a market research company were randomly assigned to a group who reviewed the draft material or to one of two control groups (Phase 3). Next, ten think-aloud interviews were conducted with patients (Phase 4). Four community consultants then met and provided their input on the materials (Phase 5). The animation script was revised after each of these phases. The draft material was then ready to hand over to the partner animation company, who created the animation (Phase 6). This was done in an iterative process with the investigators over six months. Towards the end of this period, the first draft of the complete animation was presented to 22 community members in three focus groups (Phase 7), and changes were made to the animation content in light of their feedback. In the final month of development, the voice-over was recorded and sound effects added. The final animation was then shared directly with the public via YouTube, the video sharing website that hosts user-generated videos ([www.youtube.com](http://www.youtube.com/)), as well as via the Icahn School of Medicine at Mount Sinai (ISMMS) Institute of Personalized Medicine website (<http://icahn.mssm.edu/research/institutes/institute-for-personalized-medicine>).

**Detailed description of procedures and results**

Phase 1: Review of existing online resources

When the review was conducted in the Fall of 2011, four online resources addressing genomics or WGS were identified as useful starting points for the animation. The NIH “Help Me Understand Genetics” handbook (<http://ghr.nlm.nih.gov/handbook.pdf>) explained genetic concepts well to a lay audience, but did not address WGS, and comprised >100 pages. The NHGRI website ([www.genome.gov](http://www.genome.gov/)) provided information about genomics and WGS for lay audiences, but was text heavy. The Personal Genetics Education Project ([http://pged.org](http://pged.org/)), developed by researchers at Harvard Medical School, provided a simple explanation about personal WGS and types of information generated by WGS, but no information on basic genetic concepts. The Illumina website ([www.everygenome.com](http://www.everygenome.com/)) provided a comprehensive explanation of WGS, but did not clearly discuss results of uncertain significance that could arise from WGS. We drew on these websites when creating our initial draft content, text outline and example images about genomics and WGS.

Phase 2: Expert working group

The initial text outline and example images were presented to an expert working group in December 2011. The working group comprised nine experts from a range of backgrounds including genetic counselling, genetics, pharmacogenetics, and medicine. Revisions were made based on the expert input, and then two additional genetics experts and four opportunistically recruited lay individuals reviewed the revised text and images. Further revisions were made based on this round of feedback, to produce Draft 1 of the educational text and images.

Phase 3: Online experimental survey – pilot study

In order to pilot Draft 1 of the educational material and questionnaire instruments generally, as well as to specifically explore whether the “Genetics 101” section was necessary, an online experimental survey was conducted with individuals (n=173) recruited through an online market research company. Efforts were made to recruit participants who were of diverse racial/ethnic backgrounds, specifically equal proportions of participants who were Hispanic, African American and White non-Hispanic. 53% were female; mean age was 41 years; 41% had an annual household income <$39,000; 45% were unemployed; 21% were Hispanic/Latino, 30% African American, 41% non-Hispanic white, 8% were of other racial/ethnic backgrounds. Participants were randomly assigned to either receive or not receive a Genetics 101 section in addition to the other two sections (a description of WGS and explanations of seven categories of genetic information that may potentially be generated by WGS) of the educational material. A third group received minimal information only (4 sentences). In addition, the same two genetics experts who had previously reviewed the first draft gave feedback at this point. Revisions were made to produce Draft 2.

Phase 4: Think-aloud interviews with patients

In February 2012, think-aloud interviews were conducted with a total of 10 patients recruited through the Internal Medicine Associates (IMA) Clinic at Mount Sinai Hospital. Of the ten patients who completed the think-aloud interviews were aged 23-73 years; six were female; six African American, two Hispanic, two non-Hispanic white; four had completed some college, two a high school diploma or GED, and three grade school; all had annual incomes <$39,000, six made <$20,000 per year.

In think-aloud interviews, participants are asked to verbalize their thoughts as they read the educational materials they have been provided with. Concurrent rather than retrospective verbal reporting is used because this provides insight into individuals’ immediate thoughts and reactions. Patients also answered questionnaires before and after going through the educational material.

In early February, think-aloud interviews were conducted with the first five patients to get their in-depth feedback on Draft 2. Interviews were audio-recorded and transcribed. Concurrently, a genetics expert, a genetic data analyst, and an expert in computational biology provided feedback on the “Ancestry”, “Genetic Variation,” and “DNA Variants of Uncertain Significance” sections.

Revisions were made to the Draft 2 text and images based on the feedback from the five patients and three experts, and then a new Powerpoint presentation was produced (Draft 3). In late February, the second five patients reviewed the WGS text and images. For these five think-aloud interviews, the patients viewed a flip-chart version of the Draft 3 Powerpoint text and images, and simultaneously had the text read aloud to them by the researcher conducting the interviews. Patients also answered questionnaires before and after being taken through the flip-chart. The interviews were audio-recorded and transcribed.

In the qualitative part of the interviews, some participants were confused by the relationship between DNA, chromosomes and cells. Two participants said they didn’t understand what proteins are or do. Efforts were made to revise and improve the clarity of these sections. The meaning of the term “standardized reference sequence” was particularly unclear to participants, and so this section was revised. Some participants didn’t understand how WGS could provide information about ancestry, and so the following example was added to the text: *“For example, some variants are found in people whose ancestors lived in Asia. If you have these variants, it’s likely you have ancestors that lived in this part of the world.”*  Additionally, participants expressed a need for greater clarity regarding “DNA variants of unknown significance”*,* so the following sentence was added: *“Scientists are not currently sure what role these variants play in your health.”*

In early March 2012, we obtained feedback on the “Limitations”, “Benefits” and “Categories of Information Type” sections from an academic computer engineer with expertise in genomics.

Overall, four participants stated they would like to receive this information material in the form of an animation, while three said they would like to receive it from their doctor. Further revisions were made based on the feedback from these five patients and the genomics expert, and Draft 4 was produced.

Phase 5: Meeting with community consultants

Four community consultants from the East Harlem and Upper East Side areas of Manhattan were recruited to provide their input on Draft 4. These community consultants had either worked as leaders in their communities or previously participated in another study, Community VOICES. They reviewed the Draft 4 script and images in the revised Powerpoint presentation, and were asked to give their feedback. For example, they were asked to provide input on genetic terms that they found confusing, and to comment on the content keeping in mind how people in their communities might respond to or understand the information being communicated. The meeting was audio-recorded and transcribed. No quantitative data were collected. The four community consultants provided important insights that led to several key changes to the educational material. For example, when reviewing the introductory basic genetic information (Genetics 101), they indicated that the section on variation between individuals needed to be explained more clearly, and so revisions were made to address this. This section was made clearer by presenting a pie chart with the 1% ‘spilling out’ and showing examples of things influenced by this variation, such as heart disease risk, eye color, and height. They also felt the section explaining the relationship between DNA and proteins could be clearer, and so this section was further revised to include the explanation that, *“A change in a gene could lead to a change in a protein which could affect, for instance, how your heart functions.”* In the “Making your decision” section the consultants all felt that the ‘cons’ of WGS were too frightening. We therefore replaced the words “anxious” and “depressed” which evoked specific emotions with a more general statement that potential risks may include “social and emotional consequences” of the information. They felt there needed to be greater reassurance about confidentiality, and there was discussion about the distrust that some communities have about research, particularly genetic research. Revisions were therefore made, and a statement was added that researchers would take all necessary precautions to prevent any loss to privacy. Once all revisions had been made based on the community consultants’ feedback, Draft 5 was produced.

Phase 6: Partnership with animation company

A Manhattan-based company called “The Studio,” transformed the Draft 5 script and example images into an animation in an iterative process with regular meetings with the study investigators over a six-month period.

Phase 7: Focus groups

When the first complete animation was produced, three focus groups with a total of 22 patients were conducted at Mount Sinai Hospital to obtain patients’ feedback. At this point, there was no audio for the animation, so the voice-over was spoken by one of the study investigators in the room. The focus groups were audio-recorded and transcribed. In addition, participants completed questionnaires at the beginning and end of each focus group. Changes to the animation were made in response to feedback from the focus group participants. The three focus groups each comprised 6-8 participants (22 total) aged 23-55 years. Nine were female; seven African American, six Asian, four Hispanic, four non-Hispanic white, one Native Hawaiian; 16 were employed; six made <$20,000 a year. In the quantitative questionnaires, participants’ self-rated understanding of the terms “genome, “gene”, “DNA and “chromosome” were significantly higher after compared to before viewing the animation. Twenty of 22 felt the animation was easy to understand; 15 agreed it covered information they wanted to know; and 15 were satisfied with the visuals. These positive reactions were echoed in the qualitative portions of the focus groups, with participants generally expressing that the animation was interesting and communicated the information in a way that was easy to understand. However, there was also a desire for greater emphasis on the potential negative emotional implications of WGS results, some participants said there should be more emphasis on the pros and cons of WGS, and some that they wanted a section saying where to go for more information. Participants also reported that in places the graphics competed with the audio for attention, and that it was hard to attend to the audio because too much was happening on-screen. Two sections in particular were highlighted negatively in all three focus groups. First, the section describing the types of personal results that could arise from WGS included a scene in which the main character was taken to hospital in an ambulance. Participants responded negatively to this scene, and so it was removed and replaced with a softer image (the character simply walking from one scene to the next). Second, the limitations section included a scene in which the letters of a DNA sequence floated in a teacup to represent their being interpreted, like a fortune teller interpreting tea leaves. This scene also received negative feedback, and so was replaced with a simpler (the timeline gradually turned into a DNA sequence). Both changes were made so as to distract less from the narration and provoke less negative responses.

Phase 8: Final edit

Minor revisions were made to the animation in the final month of development between the animation company and the study investigators. The voice-over was then recorded by a professional voice artist in a recording studio and sound effects were added.

**PRELIMINARY EVALUATION OF THE FINAL ANIMATED VIDEO**

Of 865 individuals who completed the post-implementation online experimental survey, two were excluded because they reported they were <18 years old, and one because they reported an unrealistically high age of 100 years and a response pattern that suggested they had not read the questionnaire. This produced the final sample size of 862 survey respondents, of whom 281 were asked to view the animated video (the Video-Information Group).

**Statistical analyses**

Within the Video-Information Group, socio-demographics, satisfaction, and informed decision-making (interest in receiving personal WGS results, DCS scale, and DCS subscales) were described using frequencies, means, and standard deviations; changes in objectively-assessed knowledge were assessed using the Wilcoxon signed-ranks test; and changes in self-rated knowledge were assessed using the McNemar test. Comparisons between the Video-Information, Written-Information and No-Information Groups were calculated as follows. **Socio-demographics** were compared between-groups using chi-square tests. **Satisfaction** with the information was compared between the Video-Information and Written-Information Groups using Kolmogorov-Smirnoff tests for the non-normally distributed continuous variables, and chi-square tests for the three categorical variables. The satisfaction measures were not assessed in the no-information group, and so these comparisons were conducted only between the two groups, the animation-information and the written-information group. **Informed decision-making** variables (interest, the DCS scale and the DCS subscale) were compared between the three experimental groups using Kruskall-Wallis tests (there were no differences between men and women so gender was not included as a covariate in these analyses). To compare changes in **objectively-assessed knowledge** between the three experimental groups while controlling for gender, analyses of covariance (ANCOVAs) with gender and objectively-assessed knowledge (pre-intervention) as covariates were conducted. Pre- and post-intervention scores were also compared cross-sectionally between the three experimental groups using Kruskall-Wallis tests. To compare changes in **self-rated knowledge** between the three experimental groups while controlling for gender, binary logistic regressions were conducted with experimental group, self-rated knowledge (pre-intervention) and gender entered as independent variables. Pre- and post-intervention scores were also compared cross-sectionally between the three experimental groups using chi-square tests. P-values <0.05 were considered significant. All analyses were performed using IBM SPSS statistics 20 (Chicago, IL).
